# Prediction of the effect of formulation on the toxicity of chemicals†
†Electronic supplementary information (ESI) available: KNIME workflows of the model building process. See DOI: 10.1039/c6tx00303f


**DOI:** 10.1039/c6tx00303f

**Published:** 2016-10-31

**Authors:** Pritesh Mistry, Daniel Neagu, Antonio Sanchez-Ruiz, Paul R. Trundle, Jonathan D. Vessey, John Paul Gosling

**Affiliations:** a Artificial Intelligence Research Group , Faculty of Engineering and Informatics , University of Bradford , Bradford , UK; b Lhasa Limited , Granary Wharf House , 2 Canal Wharf , Holbeck , Leeds , LS11 9PS , UK . Email: jonathan.vessey@lhasalimited.org; c School of Mathematics , University of Leeds , Leeds , UK

## Abstract

Two approaches for the prediction of which of two vehicles will result in lower toxicity for anticancer agents are presented. Machine-learning models are developed using decision tree, random forest and partial least squares methodologies and statistical evidence is presented to demonstrate that they represent valid models. Separately, a clustering method is presented that allows the ordering of vehicles by the toxicity they show for chemically-related compounds.

## Abstract 2

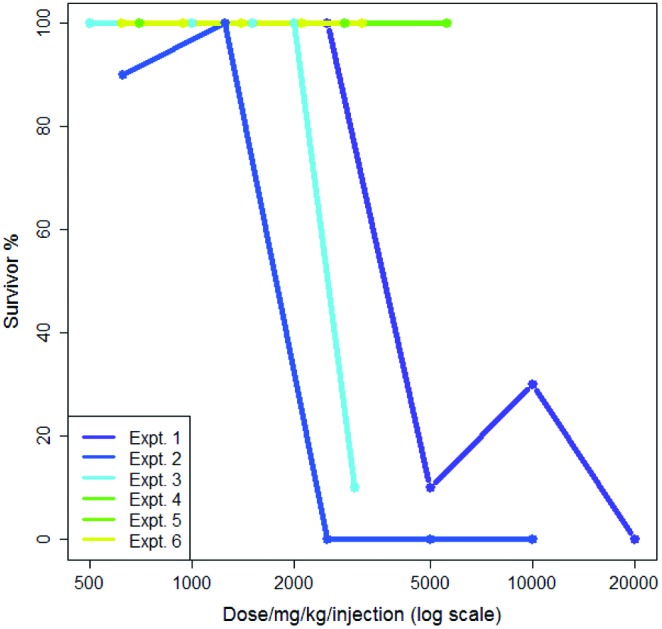
Two approaches for the prediction of which of two vehicles will result in lower toxicity for anticancer agents are presented.

## Introduction

When considering the formulation of a drug compound, many factors must be taken into account: maintaining the efficacy is one of the most important, but for some classes of drug compounds formulation to reduce toxicity becomes important too. This is particularly true for cytotoxic compounds where several formulation strategies to reduce toxicity have been used.^1^–^6^

Perhaps the simplest change in formulation is to change the dosing vehicle. Investigations into the effect of vehicle on toxicity have been done in the past. For example, differences in halocarbon toxicity using corn oil or an aqueous Emulphor vehicle have been investigated by several groups^7^–^11^ with differences in toxicity due to the vehicle also being dependent on the type of toxicity – developmental, hepatotoxic and renal – as well as the dose. Similarly, aliphatic nitrile compounds have been investigated: Farooqui *et al.*^12^ showed that the toxicity of unsaturated aliphatic nitriles in Sprague-Dawley rats was reduced by using corn oil, safflower oil, mineral oil, olive oil or Tween-20 rather than saline. In contrast, Ghanayem *et al.*^13^ found that administration of methacrylonitrile in safflower oil was more toxic than in water.

The prediction of the toxicity of chemicals using machine-learning methods has been underway for many years^14^–^17^ and is sufficiently mature to support both freely available^18^–^21^ and commercial^22^–^25^ *in silico* models. Models are based on mechanistic rationale (expert systems) or statistical correlations, and both approaches have gained regulatory acceptance for prediction of mutagenicity of genotoxic impurities.^26^

In an earlier paper,^27^ we described the prediction of the effect of the dosing vehicle on toxicity; repurposed data from the United States National Institute of Health (NIH)^28^ was used to generate dose-survival curves for drugs administered using either saline or carboxymethylcellulose (CMC), and it was found that machine-learning (ML) methods could correctly classify compounds as having lower toxicity when administered with one of the two vehicles.

In this paper, we consider how to demonstrate that the relationships that we found previously can be considered to be statistically significant and use the same approach to establish models for other pairs of vehicles.

As data for even a single compound tested using two different vehicles, with other factors being kept the same, are rare, we have also investigated how clusters of compounds containing similar chemical groups show a difference in toxicity for vehicle pairs, allowing the building of sets of vehicles ordered by their relative toxicity when used as vehicles for compounds in the cluster.

## Background

### The dataset

The dataset that was used is described in detail in our previous publication.^27^ The data have been collated over many years between the 1950's and 1980's by the National Cancer Institute's Developmental Therapeutics Program (DTP).^28^ The dataset was created to record the effect of drug compounds on animals that had been inoculated with a cancer cell line. Experiments were done using sets of, typically, 6–10 animals with variations in the dosing regime. Toxicity was measured by considering the survival rate of the animals in the test on a particular day. It is assumed that the death of the animals is due to the administered compound rather than the cancer cell line due to the short time span of the experiments, typically a few days.

The dataset consists of >2 M dose-toxicity data points; these are generated from >220k different compounds tested in ∼50 different vehicles. There are experiments on ∼40 different species represented in the dataset with the drug compound administered by ten different routes.

The dataset is free to download with explanatory instructions.^28^

### The approach to measuring differences in toxicity

It was hoped that the dataset would contain records where the only difference between two experiments was the vehicle with which the compound was administered and that it would be possible to measure a difference in toxicity where this was the case. The toxicity was measured by the number of surviving animals on a specified day. The difficulty in measuring the difference in toxicity due to the vehicle was that relatively few experiments were conducted with all other factors being the same. In order to make a comparison between experiments, therefore, a judgement was made about which factors must be the same and which might be allowed to vary; the following factors had to be the same for experiments to be considered comparable: administered compound, route of administration, host species, number of injections, injection interval, first injection day, the number of repetitions, the day on which the toxicity was assessed and any restart days. Where these factors were the same, and the vehicle was also the same, the experiments could be combined into an aggregate for these conditions. In most cases (18 992 out of 26 424 for compounds tested in either saline or CMC), there was only one set of dose-survival experiments done in a compound-vehicle combination; in other cases, however, there were more. This means that – for these aggregated data – there was both inter- and intra-lab variability, both of which are difficult to quantify.

**Fig. 1:**
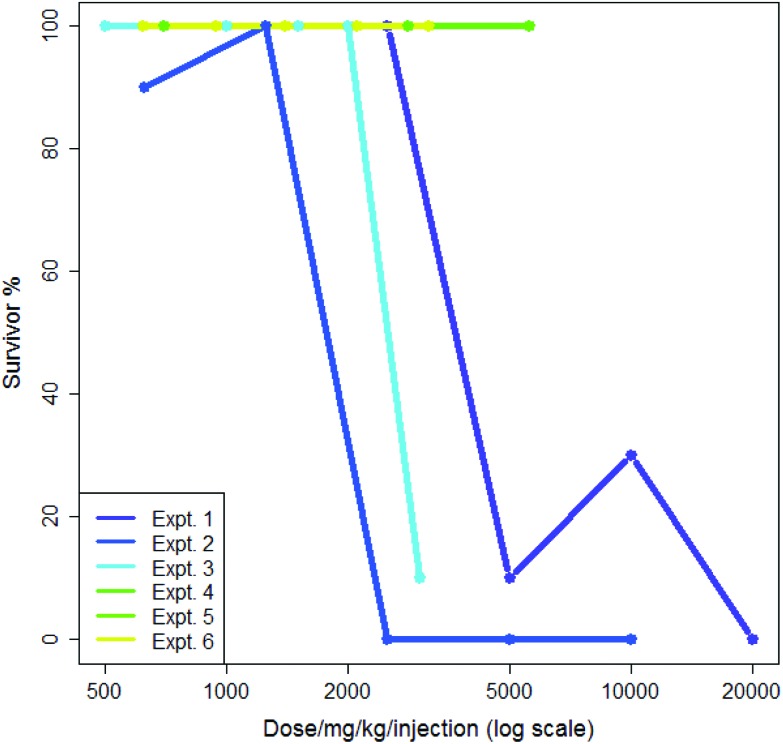
Dose-survival plot of six experiments involving single dose intraperitoneal injections of 4-di(2-chloroethyl)aminophenylalanine hydrochloride into B_2_D_6_F_1_ (BDF_1_) mice in CMC by the same screener.

5-Flurouracil is an anti-cancer agent which was tested many times in the dataset, usually administered in saline and at doses where a typical death rate was zero; this allows some measure of both intra- and inter-lab variability. In 1985 different sets of experimental conditions where more than one experiment was performed by the same screener, 1687 (85%) showed a median survival rate of 100%. Aggregating these to eliminate differences from the screener gave 1576 different sets of experiments where more than one experiment was performed of which 1325 (84%) showed a median survival rate of 100%. This suggested that both the intra- and inter-lab variability rates were low and were quite similar. Nevertheless, some variability was observed: intra-lab variability is demonstrated in Fig. 1 where six experiments involving the nitrogen mustard *para*-di-(2-chlorethyl)-aminophenylalanine hydrochloride in CMC being administered by a single intraperitoneal injection by the same screener are shown. Each experiment is compared to its own control; experiments each lasted a single day. The dose range in Fig. 1 is plotted logarithmically for purposes of illustration. Two of the six experiments, numbers 5 and 6, show exactly the same results. Three of the six experiments demonstrate high toxicity in doses over *ca*. 2500 mg per kg per injection. In the analysis in this paper, the mean value of survival at each dose would be included in the training data for the model.

**Fig. 2:**
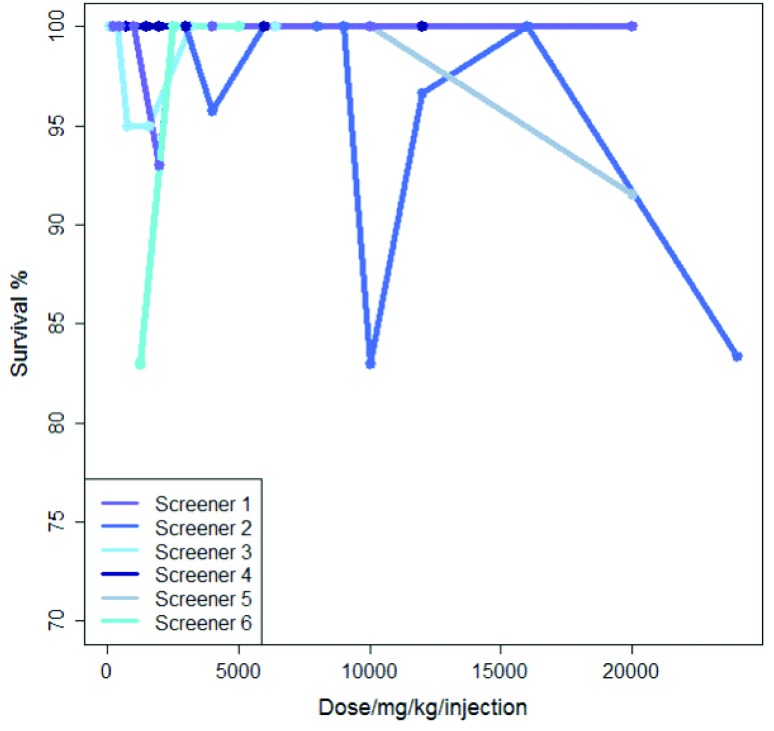
Dose-survival plot of six experiments by different screeners where mercaptopurine was administered to B_2_D_6_F_1_ (BDF_1_) mice, intraperitoneal, with a single daily injection repeated over nine days with the drug administered using saline as the vehicle.

Inter-lab variability is illustrated in Fig. 2 which shows the results from six experiments where mercaptopurine was administered to B_2_D_6_F_1_ (BDF_1_) mice, intraperitoneal, with a single daily injection repeated over nine days with the drug administered using saline as the vehicle; the dose range for each experiment varies. For these experiments there is very little variation in survival shown over the dose range, all studies showing a survival of between 83 and 100%.

**Fig. 3:**
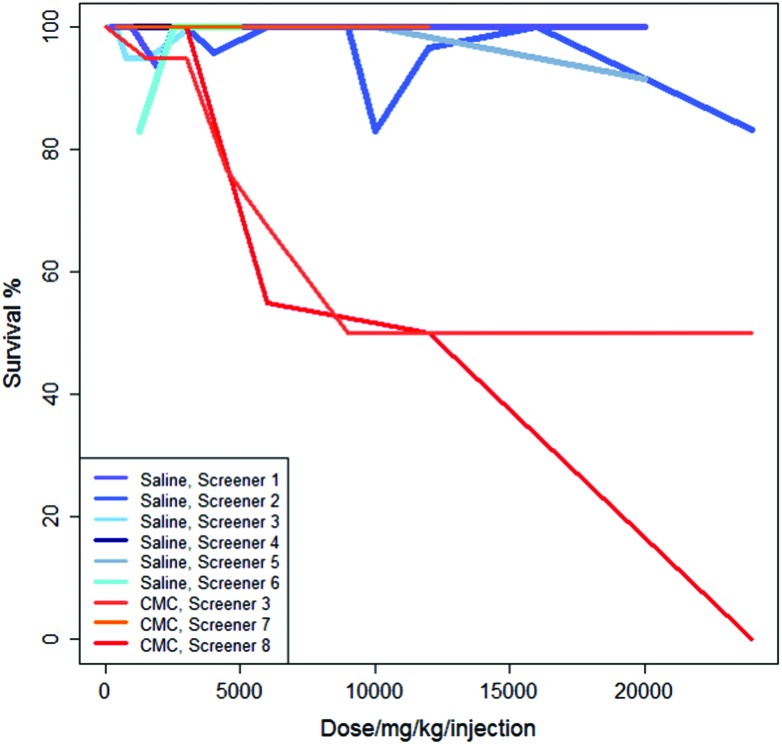
Variations in survival with dose for several experiments with mercaptopurine. Each line represents an experiment carried out by different screeners. Lines in shades of blue or grey represent six experiments where the mercaptopurine was administered in saline and lines in shades of red and orange represent three experiments where the mercaptopurine was administered in CMC.

Fig. 3 shows the studies from Fig. 2 in the context of three additional studies of the same regime but using CMC as the vehicle. For these experiments, administering the drug in saline generally results in a higher survival rate than administering the drug in CMC.

Although generally there is a decrease in survival with increasing dose, Fig. 1 and 2 show that, not infrequently, a set of experiments shows a higher survival (*i.e.* less toxicity) at a higher dose than at a lower dose: this is shown, for instance in the experiments in Fig. 1 for Experiment 1 and Experiment 2 and also in Fig. 2 for Screener 2. Such variations are commonly due to the death of a single animal from a study. In this work, such variation is considered as ‘noise’.

Where the toxicity of a compound has been tested by administering it using different vehicles and these other factors were equal, the toxicity profiles of the two experiments were compared using the area under the dose-survival curve. Where a difference in the areas under the dose-survival curve was found, the compound is considered to be less toxic when administered in one vehicle rather than the other.

As described previously,^27^ the difference in the area under the dose-survival curve (AUC) could be calculated in different ways to maximise the areas compared: the three ways considered were interpolation only; interpolation with extrapolation at the high dose end where possible and interpolation with extrapolation at both ends of the dose range where possible.

Interpolation is used where the lowest or highest doses used in two experiments differ; a point is added to the larger dose range to estimate the survival at the ‘missing’ dose thus defining a boundary for the calculation of the areas under the dose-survival curves. Extrapolation at high dose is possible where the survival has already fallen to zero and at low dose where survival at the lowest recorded dose is 100%.

**Fig. 4:**
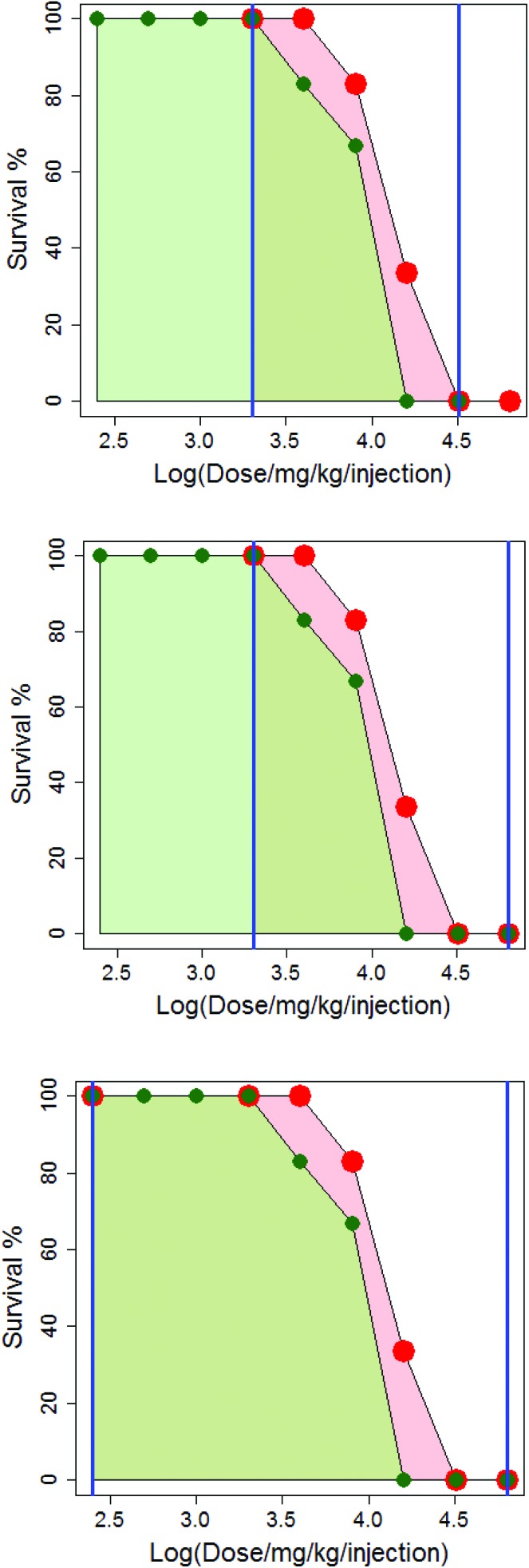
The dose-survival curves for anthracene dicarbamimidothioate (ATPU) hydrochloride administered in saline and CMC. The area under the saline curve is shaded in red and under the CMC curve in green. For the model building, the areas under the curve to be compared are shown by the blue vertical lines which change depending on whether the curves are interpolated only (top), extrapolated at high dose (middle) or extrapolated at both high and low dose (bottom).

In Fig. 4, the difference in how the areas under the dose-survival curves are calculated is illustrated for anthracene dicarbamimidothioate (ATPU) hydrochloride administered to B_2_D_6_F_1_ (BDF_1_) mice, intraperitoneal, with a single injection in either saline or CMC. The area under the dose-survival curve for the drug administered in saline is shown in pale red, while that for the drug administered in CMC is shown in green; the limits of the area under the curve are shown as blue vertical lines. In the top diagram, it can be seen that at the highest common dose, the survival rate in saline is 0% and so it is reasonable to extrapolate this survival rate to higher doses, up to log (dose per mg per kg per injection) of 4.8; this is shown in the second diagram. Additionally, as the survival rate in saline at the lowest common dose is 100% it is reasonable to extrapolate to low dose and assume the survival rate will remain the same; this is shown in the third diagram. (Note that in Fig. 4, the dose axis is shown as log (dose per mg per kg per injection). This is purely for purposes of the diagram.)

**Fig. 5:**
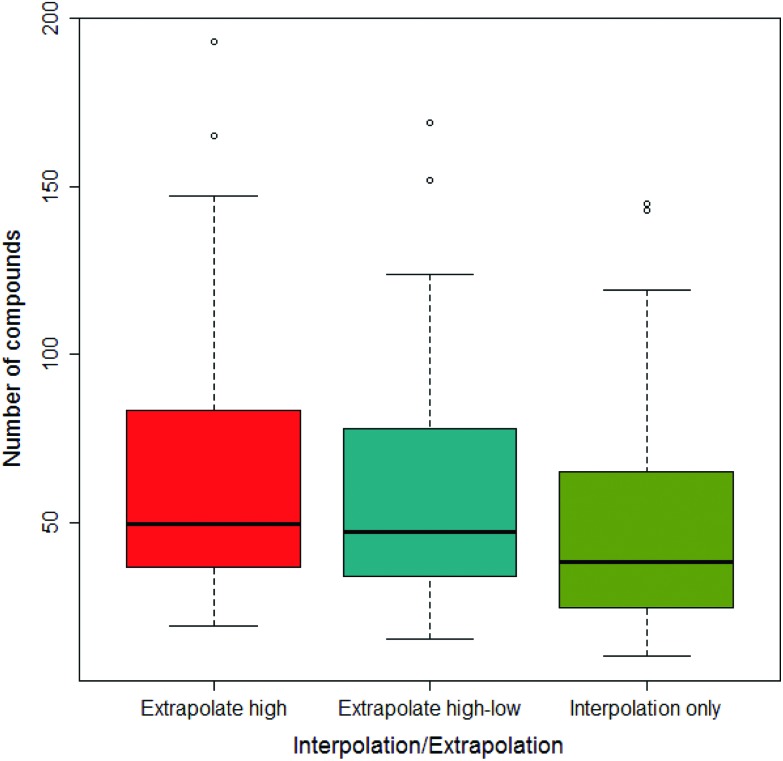
Boxplot of the variation in the number of compounds across all vehicle pairs considered to show a significant difference in the area under the dose-survival curve with the interpolation or extrapolation of data points.

Across all the drug-vehicle combinations, it was found that there was little difference in the number of compounds that showed a difference depending on the method of measuring the AUC, although when only using interpolation the median was smallest; this is shown in Fig. 5.

**Fig. 6:**
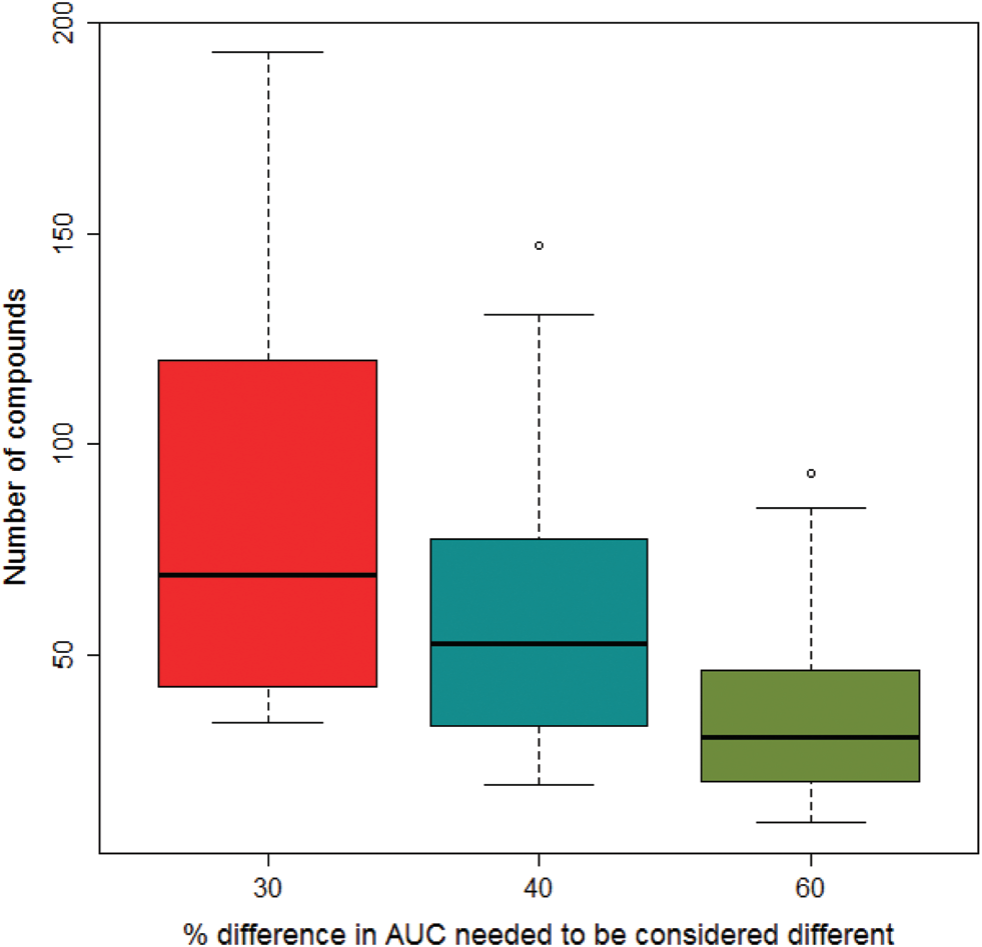
Boxplot of the variation in the number of compounds across all vehicle pairs considered to show a significant difference in the area under the dose-survival curve with the size of the difference considered to be significant.

In our previous paper we considered what would constitute a sufficiently significant difference in the area under the dose-survival curve to merit a compound being considered to be less toxic in one vehicle than another; three levels of difference were considered: 30%, 40% and 60%. Clearly the greater the difference needed to be considered significant, the fewer data would satisfy the condition. This is shown in Fig. 6 where increasing the difference in AUC needed to be considered significant reduces the number of compounds fulfilling this condition on going from 30% to 40% and 60%. In this paper, we report the results from models built separately using all three differences in AUC.

## ML model building approaches

The idea of the ML model building was to see if it was possible to build models which could classify compounds as more toxic or less toxic when administered in one of two vehicles. The classification of compounds in the training set would be made by the difference in the dose survival curve for the compound when administered in one vehicle over the other, other factors being the same. Frequently, it was found that where there were several different sets of experiments for a compound administered in two vehicles – for example a set of experiments where the host species was mouse, and a set of experiments where the host species was hamster – it might be the case that a reduction in toxicity was observed in a vehicle in one set of experiments, but not in the other. Cases where compounds did not show a consistent pattern across all the experiments for a vehicle pair fell into two types: (i) those which showed a combination of preference for one vehicle in some experiments but no preference in other experiments, which were termed equivocal and (ii) those which showed a preference for one vehicle in some experiments and for the other vehicle in other experiments, which were termed contradictory. The number of equivocal compounds was often comparable to those which showed a decided preference, whereas the number that were actually contradictory was very small. For the purposes of modelling, these equivocal and contradictory compounds together with those compounds which consistently showed no preference for either vehicle, were excluded; numbers of compounds modelled as well as the numbers excluded by being considered equivocal or contradictory are shown in Tables 1 and 2.

**Table 1:** Performance of nine models for four different vehicle pairs where the modelled dataset, *N*, was 50 compounds or more and the balanced accuracy was at or above the 99^th^ percentile of the distribution of balanced accuracies for 300 y-randomised models of the same data. Interpolation/extrapolation is how the area under the dose-toxicity curve has been treated: interpolation only means no extrapolation has taken place; high and high-low indicates that the data has been extrapolated at the high-dose or both the high- and low-dose ranges. AUC is the difference in areas under the dose-survival curve needed before it is considered significant. Equivocal is the number of compounds, not included in the modelling, which showed lower toxicity in one vehicle in some experiments but no difference in others. Contradictory is the number of compounds which showed lower toxicity for one vehicle in some experiments and for the other vehicle in other experiments. Percentile is the percentile of the balanced accuracy of the real model in the distribution of balanced accuracies for the y-randomised models. HPC is hydroxypropylcellulose (Klucel)

Vehicle pair	Interpolation/extrapolation	AUC/%	*N* (less toxic in first vehicle: second vehicle)	Equivocal	Contradictory	Modelling method	Balanced accuracy	Percentile
Saline *vs.* CMC	Extrapolate high-low	40	123 (69 : 54)	100	14	PLS	76%	99.7
Saline *vs.* CMC	Extrapolate high-low	40	123 (69 : 54)	100	14	DT	69%	100
Saline *vs.* CMC	Interpolation only	60	66 (43 : 23)	55	2	PLS	83%	99.0
Saline *vs.* CMC	Extrapolate high-low	60	79 (49 : 30)	76	4	PLS	85%	99.7
Saline *vs.* HPC	Extrapolate high-low	30	116 (69 : 47)	42	3	PLS	80%	99.3
Saline *vs.* HPC	Extrapolate high	30	131 (72 : 59)	44	3	DT	69%	99.7
Saline *vs.* saline with Tween-80	Extrapolate high-low	30	165 (92 : 73)	81	9	PLS	75%	99.0
HPC *vs.* saline with Tween-80	Extrapolate high-low	30	107 (56 : 51)	45	7	PLS	86%	99.7
HPC *vs.* saline with Tween-80	Extrapolate high	30	116 (59 : 57)	45	8	PLS	86%	100

**Table 2:** Summary of performance metrics and significance measures for 100 RF models of each of 14 combinations of vehicle pairs, interpolation/extrapolation and AUC significance. Explanation of interpolation/extrapolation, AUC, *N*, equivocal and contradictory is given in Table 1

Vehicle pair	Interpolation/extrapolation	AUC/%	*N* (less toxic in first vehicle : second vehicle)	Equivocal	Contradictory	Mean balanced accuracy/%	Mean probability of real model being better than random/% (SD)	Mean overlap between real and random models (SD)
Saline *vs.* Water	Extrapolate high	30	50 (29 : 21)	54	12	73	91 (20)	9 (16)
Saline *vs.* saline with Tween-80	Extrapolate high-low	40	119 (62 : 57)	53	6	63	85 (23)	16 (18)
Saline *vs.* MC	Interpolation only	30	60 (39 : 21)	19	3	69	81 (24)	17 (17)
Saline *vs.* HPC	Interpolation only	40	68 (36 : 32)	32	0	70	81 (28)	13 (16)
Saline *vs.* CMC	Interpolation only	60	66 (43 : 23)	55	2	71	94 (17)	8 (14)
Saline *vs.* CMC	Interpolation only	40	108 (68 : 40)	87	9	63	84 (27)	15 (16)
Saline *vs.* CMC	Interpolation only	30	144 (95 : 49)	106	18	62	90 (21)	12 (16)
Saline *vs.* CMC	Extrapolate high	60	92 (58 : 34)	70	6	79	100 (1)	0 (0)
Saline *vs.* CMC	Extrapolate high	40	130 (78 : 52)	99	15	63	91 (21)	13 (20)
Saline *vs.* CMC	Extrapolate high	30	164 (103 : 61)	112	26	61	89 (22)	13 (17)
Saline *vs.* CMC	Extrapolate high-low	60	79 (49 : 30)	76	4	73	98 (11)	3 (7)
Saline *vs.* CMC	Extrapolate high-low	40	123 (69 : 54)	100	14	66	94 (14)	10 (15)
HPC *vs.* saline with Tween-80	Interpolation only	40	62 (26 : 36)	32	5	71	88 (23)	7 (9)
HPC *vs.* saline with Tween-80	Extrapolate high-low	30	107 (56 : 51)	45	7	66	88 (23)	7 (12)

The descriptors for the model were global physico-chemical parameters such as log *P* or molecular weight as well as other simple descriptors such as MACCS keys.

In the previous paper, we reported models built using decision tree (DT) and random forest (RF) approaches; in this paper, we also report the results of models built using a Partial Least Squares (PLS) approach.

PLS is commonly used when there are a large number of descriptors compared to the number of data points. It is also a quantitative modelling approach, although refinements such as PLS-Discriminant Analysis (PLS-DA)^29^ have been developed for classification models. In the models reported in this paper, a value of 0 or 1 was assigned to the preference of one vehicle over another – for instance 0 would represent less toxicity in CMC and 1 less toxicity in saline. The PLS model gave a numerical value for the predicted vehicle with lower toxicity that was assigned to a category (0 or 1) based on whether it was greater than or less than 0.5. The approach has been used successfully by others using PLS as a classification model.^30^

In all three approaches, the models were built using a 10-fold cross-validation approach. The folds were built ensuring that the class ratio in the training sets matched that of the dataset as a whole. Possible descriptors were correlated with the observed classification of the compounds in the training set and the most strongly correlating were selected. For the DT and RF models the number of descriptors selected was equal to one tenth of the number of compounds in the training set. For the RF models, the random forest was rebuilt 100 times each with a different seed value, ensuring the RF models gave rise to different predictions. For the PLS models the number of descriptors was varied in the range 5, 10, 20, 50; the maximum number of components allowed in the PLS model was one-tenth of the number of data points.

In all cases, the datasets to be modelled had to consist of at least 50 compounds.

For each of the models, the metric that was used to measure performance was the balanced accuracy of the classification. In practice it was found that the datasets being modelled were quite balanced with all biases being 2.2 : 1 or less.

### Statistical approach to validating the models

When considering if the results from a model are significant, there are different tests that need to be passed: are the results from the model better than those that might be expected from building a model where there is no relationship between the feature being modelled and the descriptors used to build the model; is there a rationale for why the descriptors used in the model would be able to predict the activity modelled?

In this paper, the models are subjected to a rigorous statistical analysis to demonstrate that they do indeed satisfy the criteria for being considered statistically significant.

To this end, for each DT and PLS model where we wanted to confirm that the prediction could not have occurred by chance, 300 models were built using a y-randomisation process^31^ (also known as target shuffling) in which the datasets from which the models were built had the preference for one vehicle over another randomised; descriptors were then selected by correlation with the randomly assigned preference and models built as previously.

**Fig. 7:**
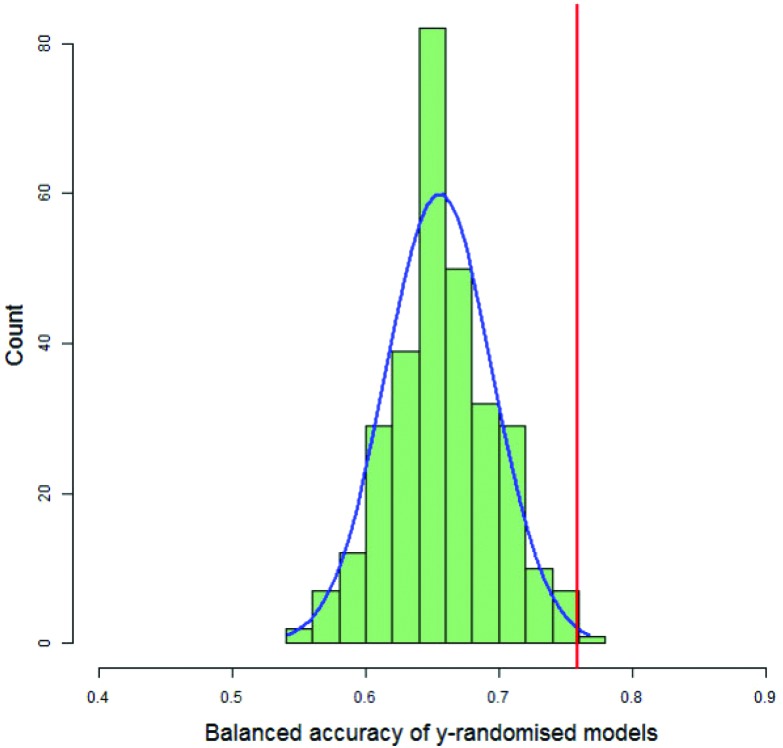
Histogram of the balanced accuracies of y-randomised PLS models for the classification of 123 compounds as having their toxicity reduced by either saline or CMC, in green. The blue line represents a normal distribution having the same mean and standard deviation as the distribution of balanced accuracies from the y-randomised models. The red line indicates the balanced accuracy of the model built with the real classification of the 123 compounds.

For each DT and PLS model, the value of the balanced accuracy of the real model was compared with the distribution of balanced accuracies from the y-randomised models. The balanced accuracy of the real model was considered to show a statistically significant difference to the distribution of the balanced accuracies of the y-randomised models if it were in or above the distribution's 99^th^ percentile, as calculated by the empirical cumulative distribution function. Fig. 7 shows a typical histogram of the distribution of balanced accuracies of 300 y-randomised PLS models for the classification of 123 compounds as having decreased toxicity in either saline or CMC where the area under the dose-survival curve has been calculated by extrapolating in both the high and low dose regions and a difference in the AUC of 40% was needed in order that the toxicities in the two different vehicles was considered different. Superimposed on the histogram is a normal distribution with the same mean and standard deviation as the distribution and the value of the balanced accuracy of the real model. In this case the real model balanced accuracy is at the 99.7^th^ percentile of the distribution.

In the case of the RF models, 100 y-randomisations were done and each y-randomisation was modelled 100 times to be comparable with the set of real RF models. The question of difference of the RF models from random thus involved comparing the distribution of balanced accuracies from the real model with each of 100 distributions from the y-randomised models. When comparing two distributions, it is straightforward to show whether or not they may be drawn from the same distribution using a *t*-test, but we were interested in measuring how much of an improvement over random each experiment represented. Two approaches to this were investigated.

**Fig. 8:**
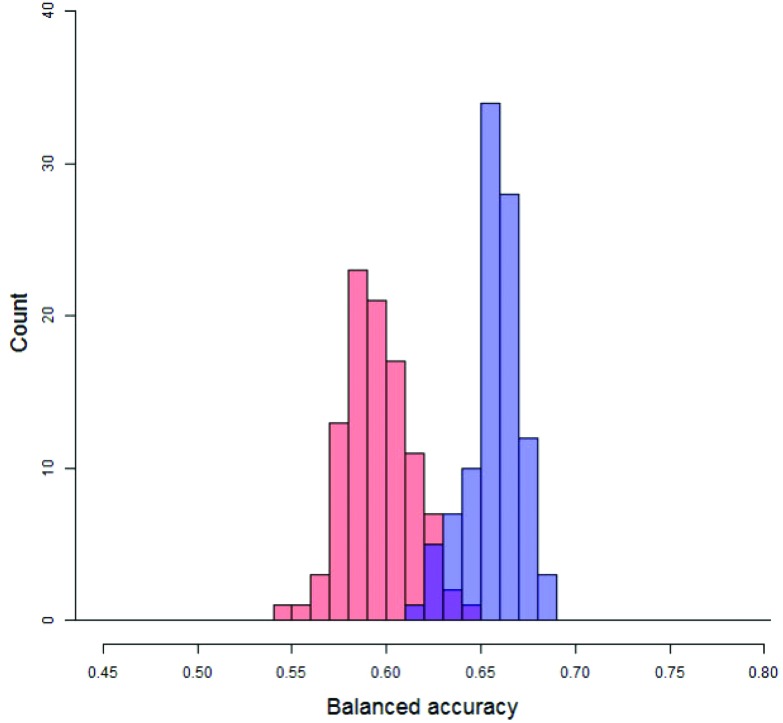
Histograms of balanced accuracy for real (blue) and one of the 100 y-randomised (red) models of 107 compounds which show a difference in toxicity between HPC and saline with Tween-80, extrapolating the dose-survival curve in both the high- and low-dose regions and using a difference in the AUC of the dose-survival curve of 30% as showing a difference in toxicity. The purple area indicates the overlap of the two distributions.

Firstly, quantifying the overlap, as shown in Fig. 8, between the distribution of real balanced accuracies and each of the distributions of y-randomised balanced accuracies. An approximation to the overlap proportion for the two distributions was done by (1) partitioning the balanced accuracy and producing histographic density estimates over the partition for both of the distributions and (2) adding up the minimum density for each partition. For completely separate distributions the overlap would be zero whilst for a situation where the real and random models produced identical distributions the overlap would be 100%. The distributions of the y-randomised models for 45 different combinations of vehicle pair and modelling conditions showed a fair amount of consistency, with median values between 55% and 64%; as a result, real models with a low performance (*i.e.* models which would fail the validation process) could still show a low overlap – so it was important to measure only those cases where the median of the distribution of balanced accuracies from the real models was greater than that of the y-randomised ones. In this study, the value of the overlap is recorded but not used by itself as a discriminating value for the significance of the experiment. The overlap coefficient remains popular for comparing two population distributions in many fields.^32^,^33^

The second approach to measuring the improvement of the real model over the set of y-randomised models involved estimating the chance that a value taken at random from the distribution of balanced accuracies of the real models was greater than one taken from each of the distributions of balanced accuracies from the y-randomised models. As this is a Monte Carlo procedure, it is also trivial to calculate standard errors for the probability estimate.

For the collection of RF models to be considered as having outperformed the y-randomised models, the mean of the above probability value had to be greater than 80%. This was an arbitrary cut-off, but there is little previous work to suggest an alternative.

## ML model building results

### DT and PLS models

The results of the DT and PLS experiments are summarised in Table 1 for those combinations of vehicle pair, interpolation/extrapolation and AUC significance where the performance based on balanced accuracy was found to be at or above the 99^th^ percentile of the distribution of balanced accuracies of the 300 corresponding models built from y-randomised data as calculated by the empirical cumulative distribution function.

As can be seen, both in quantity of models and in their overall performance the PLS modelling technique yields better results than the DT technique. Four of the combinations are for compounds where a difference in toxicity is observed when using saline and CMC, though the best performing – in terms of both balanced accuracy and improvement over the y-randomised models are the datasets of compounds where the comparison is between HPC and saline with Tween-80.

### RF models

There were 14 combinations of vehicle pair, interpolation/extrapolation and AUC significance, covering six different vehicle pairs, where the RF models were considered as significantly outperforming random.

**Fig. 9:**
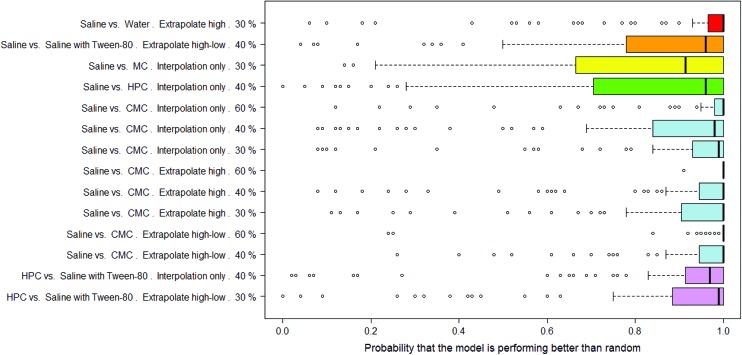
Boxplots of mean probability that a RF model outperforms a y-randomised model for 14 combinations of vehicle pair, interpolation/extrapolation and AUC significance. Experiments with the same vehicle pair are shown in the same colour.

In Fig. 9 the distributions of probabilities that the real model outperforms a y-randomised model are shown for the 14 combinations where the mean of the distribution was 80% or more. Note that the medians of these distributions tend to be 90% or more. Eight of the combinations relate to the saline *vs.* CMC pair, *i.e.* out of the nine possible combinations of interpolation/extrapolation and AUC significance for this vehicle pair, eight are considered to allow the building of RF models which outperform random. Of particular note are the results for the set of 92 compounds where, when the dose-survival curve is extrapolated only at the high dose range and a threshold of 60% is used for the difference in the AUC of the dose-survival curve, 100 RF models were built and all but one had a probability of 100% that they outperformed a model built on y-randomised data (*i.e.* all but one had zero overlap between the distributions of balanced accuracies of the real and y-randomised models).

The results in Table 2 give more details of these sets of models and it can be seen that the mean of the overlap in balanced accuracy distribution is always <20%.

Whilst considering that the models all show a performance which can be considered statistically significant, the actual balanced accuracies of the RF models are modest – in the range 61–79% – so are less than the PLS models. As with the DT and PLS models, the performances of the models for the compounds which show a difference in toxicity between saline and CMC are among the best. This suggests that not only can this difference in toxicity due to the dosing vehicle be modelled but that the descriptors used in making the models capture well which compounds will show that difference.

### RF model analysis

The performance of the models and the demonstration of their statistical significance suggests that they merit some investigation to see if the descriptors that are found to be significant can be rationalised.

One of the main reasons for variation in formulation – including variation in administration vehicle – is ensuring the drug compound is held in solution or in an emulsion or gel and so prevented from precipitating. For instance, cellulose-derived vehicles are thought to form a complex with the drug where the vehicle encapsulates the drug compound and therefore will change not only the solubility but the distribution of the drug compound.

There are a few studies where the effect of vehicle on toxicity is understood, for example nitrogen mustards have been used as cytotoxic drugs and are known to have their toxicity (and anti-tumour activity) reduced in acid media^34^,^35^ due to the protonated nitrogen being unable to form the reactive aziridinium ion.^36^ However, as far as we know, there is little systematic work rationalising the effect of vehicle on toxicity and it is hoped that this work might help in such a study.

To that end a set of 100 RF models of the whole dataset (rather than cross-validation subsets) for classifying compounds as less toxic in either saline or CMC using the settings of the best performing model (interpolation/extrapolation set to ‘extrapolate high’ and significance threshold set to 60%) was analysed to see which were the most impactful descriptors in the models. This study was done using RF models developed in R (which has the same RF algorithm as the KNIME Weka nodes used in the modelling reported above). The results are shown in Table 3.

**Table 3:** Most impactful descriptors in RF models of toxicity of compounds in saline or CMC. RDKit, Indigo and CDK MACCS are the sources of different descriptors

Descriptor	Saline > CMC rank	CMC > saline rank
Indigo number of heteroatoms	1	1
RDKit number of Lipinski hydrogen bond acceptors	2	4
Indigo number of aliphatic atoms	3	
CDK MACCS key 160: CH3	4	5
CDK MACCS key: 137 Heterocycle	5	
CDK MACCS key: 109 ACH2O		2
MACCS key: 120 Heterocyclic atom > 1		3

In this case there is a high degree of similarity between the descriptors influencing the prediction of lower toxicity in saline and lower toxicity in CMC. In addition, it can be seen that the descriptors reflect substructural features rather than whole structure properties such as solubility, log *P* or molecular weight (all of which were available to the RF models). The descriptors, three of which were also found to be significant in our earlier study,^27^ also suggest a relationship with the ability to form hydrogen bonds through the presence of a heteroatom/heterocycle or an explicit count of hydrogen bond acceptors. Further analysis of the descriptors is beyond the scope of this work.

In terms of application of the models, whilst the statistical analysis presented here shows that it is indeed possible to model the influence of the vehicle on toxicity, without a scientific rationale the models can only be used as a starting point for suggesting formulation strategies. There is insufficient data to attempt refinements such as species specific formulations.

## Investigation into differences shown by clusters of compounds

**Fig. 10:**
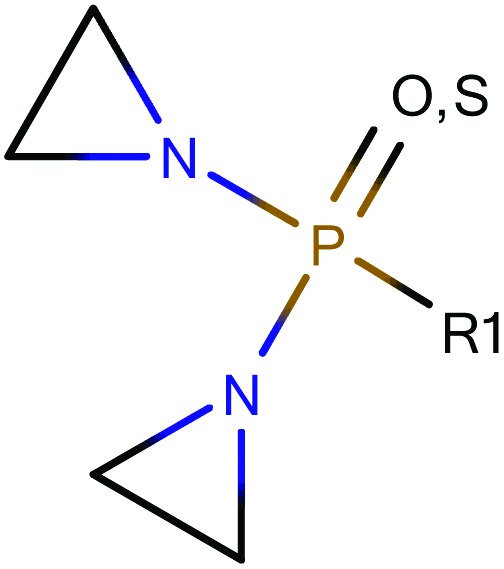
Diaziridylphosphoramide (DAP) derivatives which were seen to show a different toxicity profile in CMC than in distilled water & alcohol.

Among the datasets which contained fewer than 50 compounds, there were a few results of note. In particular, the vehicle pairs of distilled water & alcohol (DWA) *vs.* CMC all the models correctly classified groups of 11 or 14 compounds entirely correctly. The datasets that were classified correctly were all classified in the same way: compounds containing an aziridine ring were classified as less toxic in CMC than in DWA; all the compounds showing the reverse toxicity profile did not contain an aziridine ring (and, further, did not contain any other obvious common feature). Of the compounds containing an aziridine ring, many of them were diaziridylphosphoramides (DAPs) with the substructure shown in Fig. 10.

This finding suggested that a clustering approach could be taken to investigate differences in toxicity for related compounds between sets of vehicles.

Using the DAP structure shown in Fig. 10 to search the complete dataset for compounds with this substructure, 22 compounds were found for which there were data where the compound had been administered in both DWA and CMC. With such a small sample, the preference for CMC *vs.* DWA was examined with no threshold for the difference in the area under the dose-survival curve, but some rigor was introduced by considering that the area comparison had to be in the same direction however the interpolation or extrapolation were made and this was true irrespective of all other variable factors in the data, so the 22 compounds were represented by 28 sets of experiments where both CMC and DWA had been used. The areas under the curve were considered using all three interpolation/extrapolation strategies and found that a core group of 17 compounds always showed less toxicity in CMC, 4 always showed less toxicity in DWA and one always showed no preference.

It appears therefore, that drug compounds with the substructure shown in Fig. 10 might generally be formulated in CMC to reduce toxicity over a DWA vehicle.

Other differences for compounds with the substructure in Fig. 10 were investigated. For the four vehicles saline, CMC, MC and DWA, 83 compounds were found with data for at least one pair. In analysing the distribution of compounds between vehicle pairs, the approach taken was that a difference in toxicity was considered to be shown if the number of compounds with lower toxicity in one vehicle was greater than the number with lower toxicity in the other vehicle plus the number showing no difference in toxicity; where this was not the case, it was considered that the cluster of compounds showed no difference between the vehicle pair. Thus, for the 83 compounds containing the DAP structure, where the distribution between each pair is shown in Table 4, and as reported above, it is considered that the cluster shows less toxicity in CMC compared to a vehicle of DWA by 17 to 4 with one showing no difference. Similarly, the cluster shows less toxicity in CMC than in MC by 9 to 5, again with one compound showing no difference.

**Table 4:** Differences in toxicity found for clusters of compounds containing the DAP structure shown in Fig. 10, aziridines and phosphoramides. Figures in italics indicate that there is not considered to be a difference in toxicity shown by the two vehicles in the pair

Less toxic vehicle	More toxic vehicle	Preference ratio shown by cluster (less toxic : more toxic : no difference)		
		DAPs	Aziridines	NP(= [O,S])(N)(N)
CMC	DWA	17 : 4 : 1	26 : 17 : 3	14 : 2 : 1
Saline	DWA	22 : 11 : 0	30 : 18 : 3	11 : 4 : 0
MC	DWA	8 : 5 : 0	*12* : *12* : *5*	—
CMC	MC	9 : 5 : 1	19 : 12 : 3	—
Saline	CMC	18 : 7 : 9	39 : 18 : 17	*13* : *7* : *9*
Saline	MC	7 : 3 : 1	15 : 8 : 5	

**Fig. 11:**
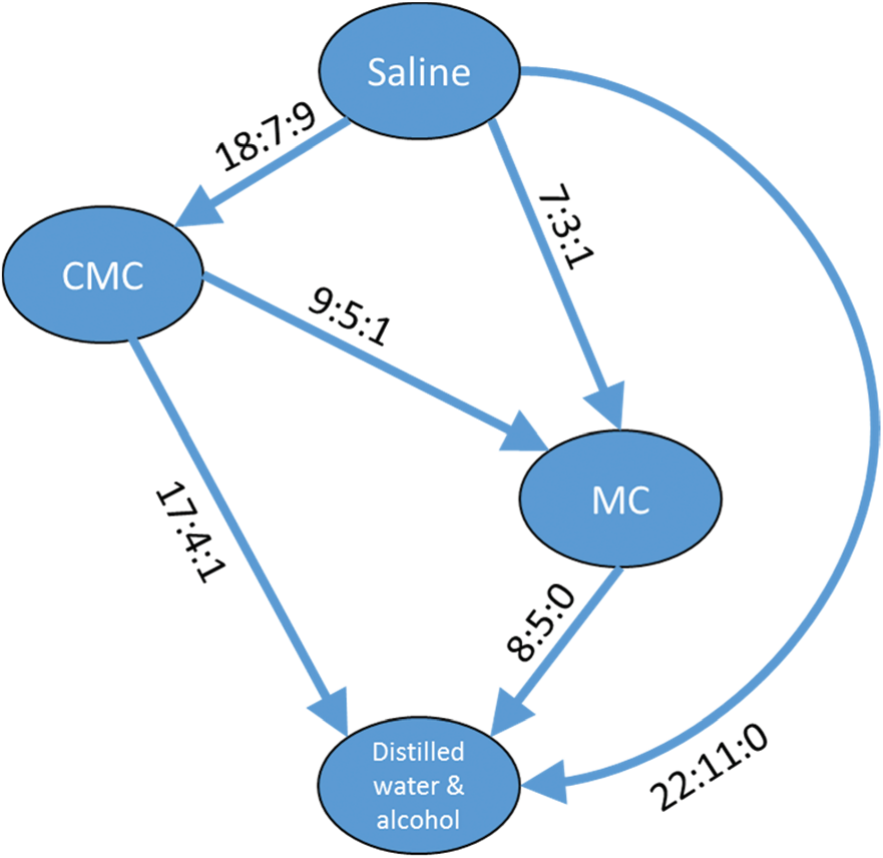
Relationships between different toxicity profiles for compounds of the DAP shown in Fig. 10. Arrows go from the vehicle with the lower toxicity to the vehicle with the higher toxicity (*i.e.* ‘safer’ to ‘less safe’). Labels on the arrows indicate the number of DAPs which are found for the vehicle pair to be less toxic : more toxic : no difference. A similar arrangement of nodes can be drawn for the aziridines and a subset of the nodes can be drawn for the phosphoramides.

The data in Table 4 show a self-consistent set of relationships in the order of the toxicity of the four vehicles with respect to compounds defined by the substructure shown in Fig. 10, which can be expressed as saline > CMC > MC > DWA where ‘>’ means ‘shows greater survival than’; the relationships are represented graphically in Fig. 11. These results could be considered to be a small rule base for choosing a vehicle for DAP drug compounds. It is important to be aware that these are trends for the cluster of 83 DAP compounds and not for individual compounds. Indeed, only one compound of the 83, ThioTEPA, had data for all six pairs, and ThioTEPA itself did not follow all the rules. Nevertheless, ThioTEPA is commonly administered in distilled water or saline.^37^,^38^

Other clusters of compounds were investigated. A related cluster of 149 compounds containing an aziridine ring showed similar relationships between the four vehicles, although as the number of compounds found to be less toxic when administered with MC rather than DWA was not greater than the number of compounds showing the reverse relationship and the number showing equality, the relationships between the four vehicles could be expressed as saline > CMC > MC = DWA, where, again, ‘>’ means ‘shows greater survival than’ and ‘=’ means ‘shows the same survival as’. In contrast, for a cluster of 38 phosphoramides defined with the SMARTS string NP(= [O,S])(N)(N) there were only enough data for the relationships between three of the vehicles (see Table 4) and the relationships were saline = CMC > DWA.

**Fig. 12:**
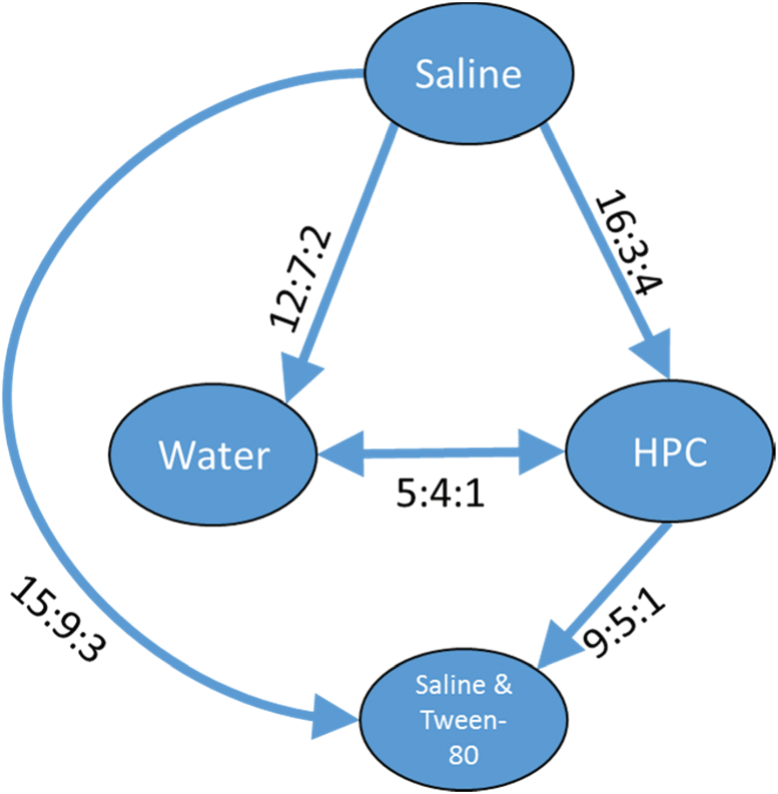
Relationships between different toxicity profiles for a cluster of 63 platinum-containing compounds. Arrows go from the vehicle with the lower toxicity to the vehicle with the higher toxicity (*i.e.* ‘safer’ to ‘less safe’). Labels on the arrows indicate the number of compounds which are found for the vehicle pair to be less toxic : more toxic : no difference. Where two or more vehicles are not considered to show a difference in toxicity, a double headed arrow is used and the label indicates less toxic on the left : less toxic on the right : no difference.

The preference ratios for the three clusters represent fully ordered sets where all the relationships have sufficient – and non-contradictory – data. For a cluster of 63 platinum containing compounds, data were found for five of the six different vehicle pairs from saline & Tween-80, HPC, water and saline. For these compounds there was insufficient data for the saline & Tween-80 *vs.* water combination. However, the five relationships which were found suggested the toxicity for platinum containing compounds varied saline > HPC = water > saline & Tween-80 and gave a full ordering of the four vehicles as can be seen in Fig. 12.

**Fig. 13:**
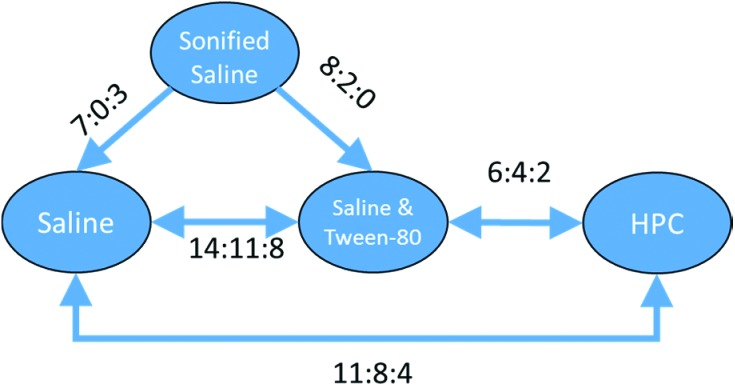
Relationships between different toxicity profiles for a cluster of 54 sulphonic acids. Arrows go from the vehicle with the lower toxicity to the vehicle with the higher toxicity (*i.e.* ‘safer’ to ‘less safe’). Labels on the arrows indicate the number of compounds which are found for the vehicle pair to be less toxic : more toxic : no difference. Where two or more vehicles are not considered to show a difference in toxicity, a double headed arrow is used and the label indicates less toxic on the left : less toxic on the right : no difference.

With a set of 54 compounds containing a sulphonic acid group, including compounds with an alkyl sulphonate counterion, with the vehicles sonified saline, saline, saline & Tween-80 and HPC, data were found again for five of the six possible relationships and were again able to form a fully order set. In this case there was no preference shown between saline, saline & Tween-80, and HPC so the relationships can be summarised as sonified saline > saline = saline & Tween-80 = HPC as shown in Fig. 13.

The five clusters of compounds referred to in this section, together with the vehicle shown to be less toxic from each pair for which there are data are supplied as SD files in the ESI.†

Several other experiments on clusters defined by groups such as arsenic compounds, nitrogen mustards, quinones, aryl carboxylic acids or simply multi-component compounds were also performed but resulted only in equivalency of several different vehicles, or single vehicle pair relationships which could not be put in a wider context. There were also cases where inconsistent relationships were recorded for example among a cluster of nitrogen mustards the inconsistent relationships HPC > saline & Tween-80, CMC = HPC, CMC = saline & Tween-80 were found. Nevertheless, the self-consistent sets of relationships found in the discussion above suggests the approach has some merit.

## Experimental work

The dataset was provided by the National Institute of Health's Developmental Therapeutics Program^28^ and was curated as described previously^27^ to give a dataset of 2 297 845 records relating to 221 656 drug compounds. 52 different vehicles were considered giving 1326 unique vehicle pairs.

All modelling and clustering were done with KNIME version 2.12.2. In this environment, descriptors were obtained from the RDKit and Indigo descriptor nodes and CDK MACCS fingerprints node; PLS models were built with the Weka 3.6 nodes, RF models were built with Weka 3.7 nodes and DT models were built with the KNIME base nodes. Statistical analyses were performed using R nodes running version 3.0.3 of R sub versioned 201508240951. Chemical substructure searches were performed using the RD Kit Substructure filter.

KNIME workflows representative of the experiments reported in this paper are available as ESI.†

## Conclusions

We have shown that models can be made for classifying which of a pair of vehicles for a drug compound can result in lower toxicity.

We have demonstrated that the approach works for several pairs of vehicles, and that a statistically rigorous evaluation of the results demonstrates that they have not come about by mere chance. We find that models built using PLS techniques give better predictive performance than those built with RF or DT methods.

We have also presented a method of ordering the relative toxicities shown by vehicle pairs for a series of clusters which generally lead to self-consistent ordered sets of vehicles.

## Conflict of interest

There are no conflicts of interest to declare.

## Supplementary Material

Supplementary information

Supplementary information
